# Recent advances in understanding and combatting *Neisseria gonorrhoeae*: a genomic perspective

**DOI:** 10.12703/r/10-65

**Published:** 2021-08-27

**Authors:** Odile B Harrison, Martin CJ Maiden

**Affiliations:** 1 Department of Zoology, University of Oxford, The Peter Medawar Building, Oxford, UK

**Keywords:** gonorrhoea, genomics, MLST, Antimicrobial resistance

## Abstract

The sexually transmitted infection (STI) gonorrhoea remains a major global public health concern. The World Health Organization (WHO) estimates that 87 million new cases in individuals who were 15 to 49 years of age occurred in 2016. The growing number of gonorrhoea cases is concerning given the rise in gonococci developing antimicrobial resistance (AMR). Therefore, a global action plan is needed to facilitate surveillance. Indeed, the WHO has made surveillance leading to the elimination of STIs (including gonorrhoea) a global health priority. The availability of whole genome sequence data offers new opportunities to combat gonorrhoea. This can be through (i) enhanced surveillance of the global prevalence of AMR, (ii) improved understanding of the population biology of the gonococcus, and (iii) opportunities to mine sequence data in the search for vaccine candidates. Here, we review the current status in *Neisseria gonorrhoeae* genomics. In particular, we explore how genomics continues to advance our understanding of this complex pathogen.

## Introduction

*Neisseria gonorrhoeae*, the gonococcus, is an obligate human pathogen that causes the sexually transmitted infection gonorrhoea^1^. Gonococcal infection often results in severe complications, ranging from disseminated infection to salpingitis or pelvic inflammatory disease, and gonococcal infections are found to be asymptomatic in over 50% of women and associated with a significant cause of infertility^2^. Therefore, prompt diagnosis and treatment are essential for a positive clinical outcome. However, the gonococcus has developed resistance against all available classes of antimicrobials and options for the effective treatment of this infection are becoming increasingly limited^2,3^. This is particularly concerning as gonorrhoea, after *Chlamydia trachomatis*, is the second most prevalent sexually transmitted infection and is globally associated with high levels of morbidity and economic cost^4^.

Infections are diagnosed by microbiological culture (with Gram stain in men with urethritis) or nucleic acid amplification tests. In settings with limited laboratory capacity, diagnosis is often based on clinical symptoms alone, followed by syndromic management. This commonly includes the use of doxycycline, an antibiotic that targets both *N. gonorrhoeae* and *C. trachomatis*, which present with similar clinical symptoms^5^. However, treatment has become complicated by the rapidly changing antimicrobial susceptibility patterns of *N. gonorrhoeae*, raising concerns about the development of untreatable gonorrhoea. Internationally, the prevalence of gonococci resistant to most antimicrobials recommended for treatment is high. For example, the recent occurrence of failures to treat gonorrhoea with the extended-spectrum cephalosporins (ESCs) cefixime and ceftriaxone and, the emergence of gonococci exhibiting high-level resistance to all other available therapeutic antimicrobials^6–8^, have caused great concern and highlight the need for a targeted approach to limit the spread of infection and prevent transmission globally.

Understanding the causative agents of disease is paramount to curing or preventing disease, and the availability of whole genome sequence (WGS) data offers novel opportunities to do this. In particular, a large amount of *N. gonorrhoeae* WGS data have become available, allowing an increasingly forensic examination of this bacterium. The knowledge gained from these analyses strengthens our capacity to detect antimicrobial resistance (AMR) while improving our understanding of the resistance phenotype and how it evolves. This knowledge also allows the population biology of the gonococcus to be resolved, facilitating global surveillance. Finally, WGS data provide opportunities for vaccine development through the characterisation of antigenic variability. Here, we review progress made in the field of gonococcal genomics, examining how WGS data have increased our knowledge of this pathogen. In particular, we focus on advances made in the detection of AMR, the genetic determinants conferring this phenotype, and the tools developed to identify AMR. We assess progress made in understanding the population biology of this pathogen and discuss the current status in vaccine development and how WGS can support this activity. To conclude, we review the challenges that remain and discuss the approaches needed to overcome them.

## Antimicrobial resistance

In the absence of vaccines, antimicrobial therapy is critical to cure infection and limit its spread^9^. The effectiveness of antibiotics to treat gonorrhoea is becoming limited by the global emergence and spread of gonococci resistant to antimicrobials, combined with increasing rates of gonorrhoea. The total number of confirmed cases reported in 2018 in 28 European Union/European Economic Area (EU/EEA) member states was 100,673 (with an overall crude notification rate of 26.42 per 100,000 population), representing a 12% increase in the total number of reported cases compared with 2017^10,11^. In the UK alone, 61,775 confirmed cases were reported in 2018, representing a 26% increase compared with 2017^10^. In the US and its territories, 556,413 cases of gonorrhoea were reported in 2017, representing a rate of 168.9 cases per 100,000 population and a 19% increase compared with 2016^12^. In Australia, the number of confirmed cases rose from 23,875 in 2016 to 28,364 in 2017 with a rate of 118 per 100,000 population^13^. This was associated with a 63% increase in notification rates between 2012 and 2016. In China, 133,156 confirmed cases were reported in 2018, representing a rate of 10.06 cases per 100,000 population^14^. Similarly, a rate of 88.6 cases per 100,000 population was identified in 2015 in Japan^10,15^. These data are consistent with a global trend of increasing rates of gonorrhoea. However, there are differences in the reporting of incidence and prevalence data among countries, even where effective surveillance systems are in place. In addition, different jurisdictions have their own surveillance methodologies and diagnostic tests such that global estimates have to be interpreted with caution^3^. So it is challenging to assess effectively global rates of gonorrhoea prevalence.

**Figure 1.**
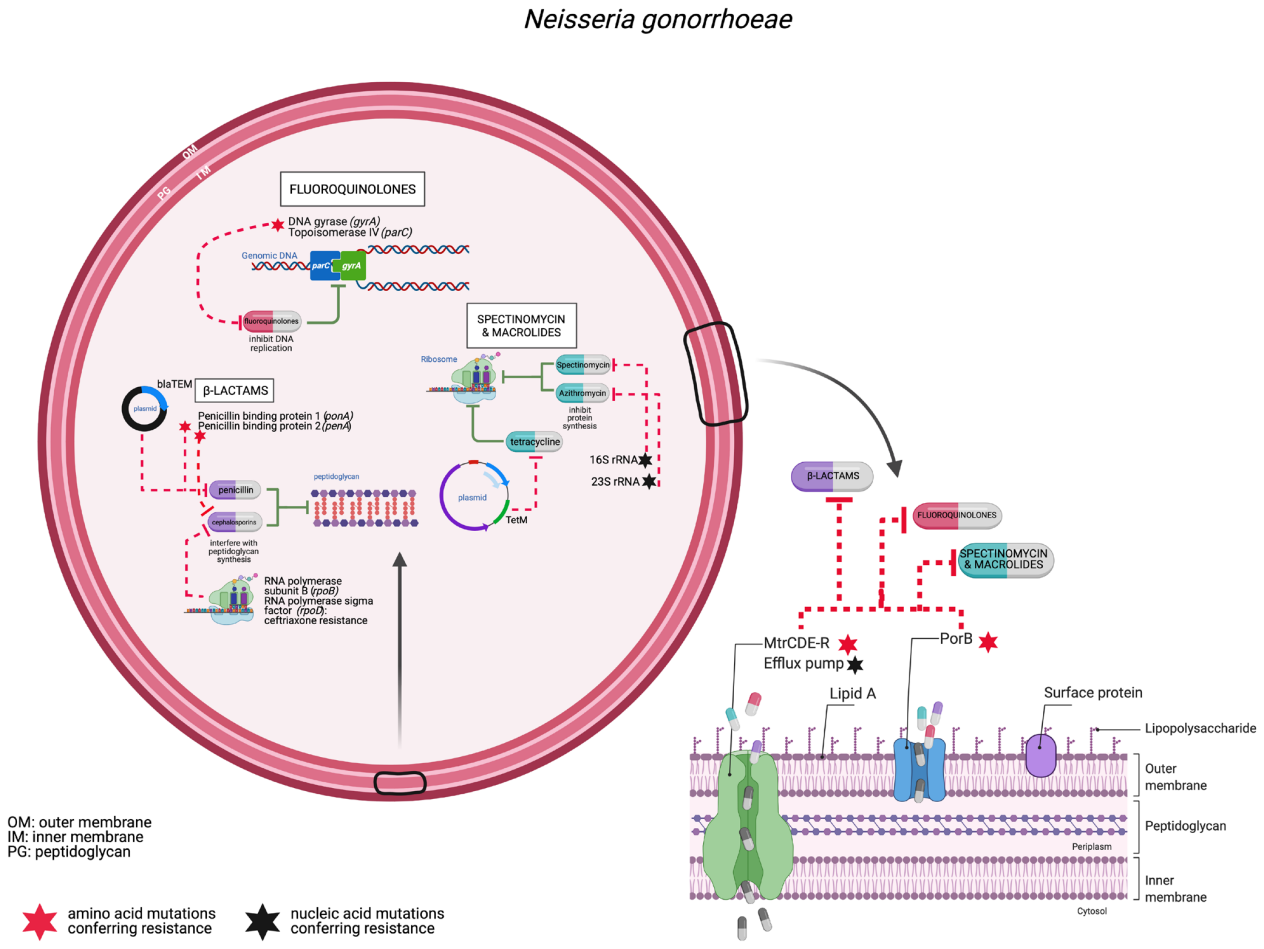
Antimicrobial resistance mechanisms in the gonococcus. Schematic figure of a gonococcus depicting chromosomally and plasmid-mediated resistance determinants. Multiple antibiotics target the gonococcus against which the bacterium has in turn developed resistance. This includes (i) fluoroquinolones (e.g., ciprofloxacin), which inhibit the activity of topoisomerases involved in DNA replication. Resistance to fluoroquinolones is conferred by non-synonymous mutations in the topoisomerase-encoding genes *gyrA* and *parC*^24,25^; (ii) beta-lactams target penicillin binding protein 1 (PBP1) and 2, which are required for peptidoglycan synthesis. Amino acid mutations in *ponA* and *penA*, which encode PBP1 and PBP2, respectively, confer resistance to beta-lactams^26–28^. Gonococci are increasingly developing reduced susceptibility to third-generation cephalosporins, including cefixime and ceftriaxone, with the presence of multiple genetic determinants required for this phenotype, including mosaic *penA* alleles^29–31^. More recently, mutations in *rpoB* and *rpoD* have been found to confer resistance to ceftriaxone only in the absence of mosaic *penA* alleles^32^; (iii) spectinomycin and macrolides, including azithromycin, bind to ribosomes inhibiting protein synthesis. Mutations in the 16S and 23S rRNA confer resistance to spectinomycin and azithromycin, respectively^33–35^. Additional chromosomally mediated resistance determinants include amino acid mutations in loop 3 of the outer membrane porin PorB, which reduces permeability, thereby inhibiting the influx of antibiotics through the porin channel^36^. Mutations in efflux pump genes *mtrRCDE*, particularly in the promoter of *mtrR*, which encodes the MtrR repressor protein, lead to overexpression and increased activity of the efflux pump complex, conferring resistance^37–39^. Mosaic *mtrD* genes have been found to confer resistance to azithromycin^40^. Plasmid-encoded resistance includes the plasmid-encoded TEM-1 penicillinase (bla_*TEM-1*_), which inactivates the beta-lactam ring of penicillin and the conjugative plasmid harbouring the *tetM* gene^19,21^. IM, inner membrane; OM, outer membrane; PG, peptidoglycan. Red stars indicate non-synonymous nucleotide mutations resulting in amino acid mutations; black stars nucleic acid mutations in the rRNA nucleotide sequences. Green lines indicate antibiotic targets; dashed red lines depict resistance mechanisms. Figure created using BioRender.

This increasing trend in gonococcal prevalence is accompanied by global reports of gonococci exhibiting decreased susceptibility to several classes of antimicrobials^2,16^. The gonococcus employs multiple genetic mechanisms to overcome the effects of antimicrobial agents and is extremely adept in developing AMR (Figure 1). Most of the genetic determinants that confer AMR in *N. gonorrhoeae* are chromosomally encoded (Table 1), with plasmid-mediated AMR associated only with the *bla*TEM gene, which confers high-level resistance to penicillin^17–19^, and the *tetM* gene associated with resistance to tetracycline^20,21^ (Table 1). Resistance to fluoroquinolones is conferred through the presence of non-synonymous mutations in DNA gyrase, *gyrA*, or the topoisomerase IV gene, *parC*, and amino acid mutations in both GyrA and ParC result in higher levels of resistance to fluoroquinolones^22,23^.

**Table 1.** Antimicrobial resistance determinants.

Antimicrobial and inhibitory effect	Described resistance mechanism and determinants	Nomenclature in PubMLST	References
**Plasmid-mediated:**			
*Beta-lactams, including penicillin (inhibits cell wall synthesis, bactericidal)*			
***blaTEM***	**Beta-lactamase plasmid containing the *blaTEM* gene encoding the enzyme beta-lactamase which** **hydrolyses the peptide bond of the four-membered beta-lactam, thus inactivating the antibiotic.** Several beta-lactamase plasmids have been defined based on their first source of geographic origin. This includes (i) p*bla*.As (Asia) (7.4 kb), (ii) p*bla*.Af (Africa) (5.6 kb), (iii) p*bla*.Ni (Nimes, France) (6.8 kb), (iv) p*bla*.NZ (New Zealand) (9.3 kb), (v) p*bla*.Rio (Rio de Janeiro, also known as Toronto/Rio) (5.1 kb), (vi) p*bla*. Jo (Johannesburg) (4.8 kb) and (vii) p*bla*.Au (Australia) (3.2 kb). Additional plasmids have been described, including the Toronto plasmid, a derivative of the Asian plasmid, and a novel Canadian plasmid variant of the Africa-type possessing a 6-bp deletion at the 5′ end of blaTEM. Several *blaTEM* alleles have been identified, including TEM-1 (NEIS2357 alleles 3 and 9), TEM-135 (NEIS2357 alleles 2 and 8, M182→T substitution) and TEM-191 (NEIS2357 allele 14 E239→K substitution).	NEIS2357 (*blaTEM*)	18,44–47
*Tetracyclines, including doxycycline (inhibits protein synthesis, bactericidal)*			
***tetM***	**Conjugative plasmid containing *tetM* gene which inactivates tetracyclines.** Several conjugative plasmids have been defined on the basis of the presence/absence of particular genes found in the plasmid. This includes pConj.1 and pConj.2, which both contain *tetM* (NEIS2210 allele 2, American); pConj.3 and pConj.4, which contain *tetM* (NEIS2210 allele 1, Dutch), and an additional plasmid identified in Uruguay with a TetM gene homologous with one identified in a meningococcus.	NEIS2210 (*tetM*)	18,20,44,48
**Chromosomally mediated:**			
*Sulphonamides (bacteriostatic; inhibit growth)*			
***folP***	**The dihydropteroate synthase enzyme, FolP:** This protein synthesises folic acid in the bacterium. Sulfonamides act as competitive inhibitors of this enzyme, thereby inhibiting growth of the bacterium. Resistance is mediated through over-synthesis of *p-*aminobenzoic acid or non-synonymous mutations in the *folP* gene. Several amino acid substitutions have been described in *Neisseria* species, including F31→L, P68→S, P68→L and R228→S.	NEIS1609 (*folP*)	49,50
*Penicillins, beta-lactams and third-generation cephalosporins (bactericidal; inhibit cell wall synthesis)*			
***penA***	**Penicillin binding protein 2, PBP2:** It is implicated in peptidoglycan biosynthesis and encodes a monofunctional transpeptidase. Amino acid mutations in the transpeptidase region are associated with reduced susceptibility. Several mosaic *penA* alleles with up to 70 amino acid alterations have been described with, in particular, mosaic allele XXXIV associated with decreased susceptibility to third-generation cephalosporins (when found concomitantly with other mutations). NEIS1753 alleles 266, 281, 498, 660, 1240, 1503, 1522, 1523, 1524, 1525 and 1526 are XXXIV mosaic alleles.	NEIS1753 (*penA*)	26,28,51,52
***mtr***	**MtrR and MtrCDE efflux pump:** The *mtrR* gene regulates expression of the efflux pump consisting of MtrC-MtrD-MtrE. Mutations in the locus results in overexpression and increased efflux from the pump. Mutations known to alter expression include a single-nucleotide [A] deletion in the 13-bp inverted repeat sequence in the promoter sequence upstream of *mtrR* (^pro^NEIS1635 allele 3) or the mutation A→C in the repeat sequence (^pro^NEIS1635 alleles 4, 5, 7, 9, 10, 11, 12, 13 and 15); an A→G nucleotide mutation 120 bp upstream of the *mtrC* start codon; a G45→D amino acid substitution in the MtrR protein and finally mosaicisms in MtrD, resulting in structural changes that enhance recognition and transport of antimicrobial agents.	NEIS1635 (*mtrR*), ^pro^NEIS1635 (*mtrR* promoter) and NEIS1633 (*mtrD*)	37,38,40,53,54
***porB***	**Outer-membrane porin PorB:** PorB is a porin that mediates ion exchange with the environment. It is essential for viability because of its ability to allow nutrient access to the periplasm. Non-synonymous mutations in loop 3 of PorB allele PIB (G120→K, G120→D, G120→N and/or A121→D, A121→ N, A121→G) reduce influx of antibiotics. NEIS2020 alleles: 204, 524, 539, 634, 792, 832, 1140, 1608, 1611, 1901, 1961, 1993, 2032, 2083, 2136, 2200, 2588, 3767, 3824, 3833, 3972 and 4304 possess G120→K and A121→D mutations associated with resistance.	NEIS2020 (*porB*)	36,55
***ponA***	**Penicillin binding protein 1, PonA:** PonA is a transpeptidase implicated in peptidoglycan synthesis. A non-synonymous mutation in *ponA*, L421→P, reduces penicillin acylation.	NEIS0414 (*ponC*)	26,27
***rpoB***	**RNA polymerase subunit B, RpoB:** The *rpoB* gene encodes the β subunit of bacterial RNA polymerase. The following non-synonymous mutation has been detected in a US clinical *N. gonorrhoeae* isolate exhibiting reduced susceptibility to ceftriaxone: R201→H. This isolate did not possess a mosaic *penA* allele. A further two mutations were subsequently detected arising spontaneously during *in vitro* experiments: P157 →L and G158 →V.	NEIS0123 (*rpoB*)	32
***rpoD***	**RNA polymerase sigma factor, RpoD:** RpoD promotes the attachment of RNA polymerase to specific initiation sites.	NEIS1466 (*rpoD*)	32
*Fluoroquinolones (bactericidal; inhibits DNA metabolism)*			
DNA gyrase and topoisomerase IV are highly conserved type II topoisomerases essential for DNA metabolism. Fluoroquinolones inhibit these enzymes, resulting in bactericidal activity.			
***gyrA***	**DNA gyrase, GyrA:** Amino acid mutations, including S91→F, D95→N, D95→G and D95 →A, decrease fluoroquinolone binding to GyrA. Alone, these mutations provide low to intermediate resistance.	NEIS1310 (*gyrA*)	23,24,25,56
***parC***	**DNA topoisomerase, ParC:** Amino acid mutations D86→N, S88→P and E91→K. When in association with mutations in GyrA, these alterations result in high-level resistance to fluoroquinolones.	NEIS0414 (*parC*)	22
*Macrolides (inhibit protein synthesis in a bacteriostatic effect)*			
**23S rRNA**	Macrolides such as azithromycin or erythromycin bind to the 50S ribosomal subunit and as a result inhibit protein synthesis. Nucleotide polymorphisms, including C2611T and A2059G in 23S rRNA, result in reduced affinity of 23S rRNA with the 50S macrolide target.	23S_rRNA	34
**MtrRCDE**	**MtrR and MtrCDE efflux pump:** Mosaic Mtr operons derived from other *Neisseria* species lead to resistance to azithromycin in the gonococcus. In particular, full-length mosaic *mtrD* genes are implicated in higher levels of resistance.	NEIS1632 (mtrE) NEIS1633 (mtrD) NEIS1634 (mtrC) NEIS1635 (*mtrR*)	40,57–59
**16S rRNA** ***rspE***	Spectinomycin binds to the 30S ribosomal subunit and inhibits protein translation. A single-nucleotide polymorphism in the spectinomycin region of 16S rRNA has been shown to result in high- level resistance to spectinomycin. Deletion of a valine at residue 25 and a K26→E alteration in the 30S ribosomal protein gene S5, *rpsE*, have been shown to confer high-level spectinomycin resistance.	NEIS0149 (*rspE*) 16S_rRNA	60

An increasing level of resistance or reduced susceptibility to beta-lactams, including penicillin and third-generation cephalosporins, is often the result of combinations of mutations in several genetic determinants (Table 1). These include (i) the presence of mosaic *penA* genes encoding penicillin binding protein 2, genetic fragments of which often are acquired through horizontal gene transfer (HGT) from commensal *Neisseria* species^41–43^; (ii) non-synonymous mutations in the *ponA* gene, which encodes penicillin binding protein 1^26,27^; (iii) the presence of mutations in the *mtrR* repressor gene or its promotor sequence, resulting in overexpression of the MtrCDE efflux pump system and secretion of antimicrobial agents^37,38^; and (iv) mutations of the outer membrane channel porin PorB variant PIB, resulting in a decreased influx of penicillin, through reduced permeability of the porin^26,36^. In addition, mutations in *rpoB*, encoding RNA polymerase subunit B, and *rpoD*, encoding the RNA polymerase sigma factor, have been identified (Table 1)^32^. These mutations, which cause large-scale transcriptional changes, including an increase in *ponA* expression, were detected in clinical isolates which lacked *penA* mosaic alleles and yet exhibited reduced susceptibility specifically towards ceftriaxone only. The number of potential mutations implicated in conferring resistance to beta-lactams highlights the genetic diversity and complexity of this genotype and the possibility that other as-yet-unknown mutations are present or indeed will arise in the future.

The macrolides, azithromycin and erythromycin inhibit protein synthesis by binding to the 23S rRNA component of the 50S ribosome. Mutations in 23S rRNA can lead to resistance and include a C2611T change resulting in low levels of resistance or an A2059G mutation leading to increased resistance^61,62^. Levels of resistance are also dependent on how many of the four copies of 23S RNA contain either mutation such that, for example, A2059G mutations, if present in three or all four of the 23S rRNA alleles, result in high-level azithromycin resistance^6,62^. Mosaic *mtr* alleles are also associated with conferring resistance to azithromycin^40,57–59^. The mechanism of resistance is distinct from those associated with increased expression of the MtrCDE efflux pump and is due to the presence of mosaic *mtr* operons where, in particular, full-length mosaic *mtrD* genes combined with a mosaic *mtr* operon lead to much higher levels of resistance to azithromycin compared with isolates with mosaic *mtr* operons alone. Such *mtr* operons were found to result from interspecies HGT with other *Neisseria* species, including *N. meningitidis* and *N. lactamica*. Finally, resistance to spectinomycin is conferred through mutations in the *rpsE* gene, which encodes the S5 ribosomal protein, and T24P mutations that induce low levels of resistance^63^. A deletion of a valine residue at amino acid 25 and a K26E amino acid substitution have also been found to be associated with higher levels of resistance^60^. Resistance to spectinomycin can also occur through a C1192U nucleotide polymorphism in 16S rRNA^33^.

In addition to the genetic mutations described above, all of which expand in response to antimicrobial exposure, selection for resistance in gonococci may occur as a consequence of indirect exposure through the consumption of antibiotics intended to treat another pathogen^64^. For example, differences in the consumption of fluoroquinolones across 24 EU/EEA countries correlated with ciprofloxacin resistance in gonococci^65^. Seasonal variations in antibiotic consumption have also been associated with fluctuations in numbers of gonococci exhibiting resistance to azithromycin, possibly a consequence of increased use of macrolide to treat respiratory infections^66^. Therefore, a driver in the development of AMR in the gonococcus may also result from the bystander effect, which may be more prevalent than anticipated, particularly since gonorrhoea infections are often asymptomatic.

To monitor resistance, gonococci are classified as either multidrug-resistant (MDR-GC) or extensively drug-resistant (XDR-GC): MDR-GC is defined as a gonococcus with decreased susceptibility to one currently recommended therapy (cephalosporin or azithromycin) plus resistance to at least two other antimicrobials, whereas XDR-GC is a gonococcus with decreased susceptibility to two currently recommended therapies (i.e., cephalosporin and azithromycin) plus resistance to at least two other antimicrobials^67,68^. Rapid detection of MDR-GC or XDR-GC is crucial to limit their spread, and advances made in genomics play an increasingly central role in assisting in this where this technology is available. For example, WGS studies identified an association between the mosaic XXXIV *penA* allele and reduced susceptibility to the ESC cefixime when found in conjunction with additional resistance factors^29^. Genomic epidemiology studies have also allowed transmission outbreaks of azithromycin-resistant gonococci to be tracked in England^69^, and further studies have elucidated transmission networks in men who have sex with men in Australia^70^. WGS studies identified mosaicisms in the *mtrD* gene (a component of the *mtrCDE* efflux pump) that enhance the capacity of the protein to export antimicrobial agents^40^.

Genomic technologies therefore offer the tantalising prospect of enhancing surveillance of AMR with several bioinformatic tools developed to predict AMR from WGS data. These include (i) Antimicrobial Resistance Identification By Assembly (ARIBA), which identifies AMR genes and variants from paired sequencing reads through the use of a combined mapping/alignment and targeted local assembly approach^71^; (ii) the Pathogenwatch AMR prediction module, PAARSNP, which can be used through the Pathogenwatch portal and which uses resistance databases developed in-house^72,73^; and (iii) ABRICATE^74^. Performance of these *in silico* AMR prediction tools is dependent on the availability of accurate and curated AMR databases such as ResFinder^75^ and CARD^76^ and those reviewed by Hendriksen *et al*.^77^. Concordance between phenotype and genotype is stronger for some genetic determinants than others. For example, ciprofloxacin resistance can be readily determined from sequence data, whereas predicting beta-lactam–resistant genotypes is more challenging, particularly as this is often an additive effect resulting from multiple mutations. As a result, several studies have encountered difficulties when using WGS to predict such AMR phenotypes^78,79^.

Other approaches have employed multivariate linear regression models to identify genetic predictors of minimum inhibition concentrations (MICs). These models predict MICs for five gonorrhoea antimicrobials that exhibit different genetic mechanisms of resistance: cefixime, penicillin, azithromycin, ciprofloxacin and tetracycline^80^. More recently, a method called ‘genomic neighbour typing’, which allows the AMR phenotype of a bacterial sample to be predicted through the identification of its closest relatives in a database of genomes which have corresponding AMR metadata, has been described. This method was tested in both *Streptococcus pneumoniae* and *N. gonorrhoeae* datasets, and preliminary results indicate that this approach may work well for the pneumococcus given the greater sequence diversity observed in its core genome, which allows specific signature sequences to be more readily detected and matched to individual lineages. However, the lower diversity found in the gonococcal core genome results in the identification of multiple related neighbours which confounds obtained signals^81^.

An alternative approach for tracking AMR in the gonococcus is through the use of a multilocus sequence typing scheme, NG STAR (*Neisseria gonorrhoeae* sequence typing for antimicrobial resistance), which indexes variability found in nucleotide sequence fragments from seven genes associated with AMR (*penA*, *mtrR*, *porB*, *ponA*, *gyrA*, *parC,* and 23 rRNA)^82^. Using an online, publicly available database (https://ngstar.canada.ca), users can submit new alleles for curation, allowing variation in genes conferring resistance to be defined and tracked. New allelic profiles are assigned NG STAR sequence types (STs), which are associated with corresponding isolate records. Since its publication, this tool has been used in several international studies assessing AMR, thus providing a tool with which to track the worldwide dissemination of resistant clones. All described genes associated with AMR in the gonococcus, both chromosomally and plasmid-mediated, have also been defined in the pubMLST.org/neisseria database with mutations annotated in allelic variants^83^. This database includes the NG STAR, multi-locus sequence typing (MLST) and *N. gonorrhoeae* multi-antigen sequence typing (NG MAST v2.0) schemes^84^. As a result, WGSs deposited in PubMLST are annotated in all of these schemes, allowing AMR to be evaluated in combination with conventional typing schemes and providing a publicly available resource for the analysis of gonococcal WGSs. Furthermore, WGSs can be directly queried for any of these schemes without the need for WGSs to be deposited. An additional publicly available tool, Gen2Epi, facilitates gonococcal WGS assembly followed by automatic retrieval of molecular epidemiological information, including NG STAR, MLST and NG MAST and AMR genotypes^85^.

## Gonococcal population genomics

The study of genomics will play an increasingly important role in enhancing surveillance of AMR and in the development of molecular diagnostic tools for the detection of this phenotype. AMR emergence has to be studied alongside population structure and genome content since the dynamics of gonococcal population biology is complex and highly influenced by HGT, which results in diversification and reassortment of genetic variation over time^86,87^.

Several molecular typing tools have been developed to identify gonococcal isolates, including (i) MLST, which indexes the diversity found at seven housekeeping gene fragments, and (ii) the NG MAST scheme, where nucleotide sequence fragments of the outer membrane proteins PorB and TbpB are used to define NG MAST STs^88,89^. MLST is based on the characterisation of fragments from housekeeping genes under stabilising selection and remains the method of choice for typing many bacterial species, including *Neisseria meningitidis*, the meningococcus, for which MLST was first developed^90^. Meningococcal MLST STs provide a convenient means for tracking the epidemiology and population biology of this bacterium since STs can be grouped into coherent groups known as ‘clonal complexes’ (ccs)^91^. A number of these ccs, ‘the hyperinvasive meningococci’, are responsible for the majority of invasive disease cases worldwide and are stable over time and global spread^91,92^. The levels of linkage disequilibrium observed in meningococci result in the non-random association of MLST allelic profiles, producing discrete persistent meningococcal lineages, some of which exhibit an invasive phenotype^93^. Studies of the genetic diversity of gonococcal housekeeping genes have shown that, in addition to diversification arising from randomly distributed point mutations, these genes are frequently subject to HGT^94,95^. As a result, some gonococci, though possessing the same seven locus MLST STs, may have different ancestry in the majority of their loci and consequently MLST STs cannot be used to reliably evaluate gonococcal populations^84,96^. In addition, combinations of MLST alleles in gonococci are unlikely to be associated with transmission fitness or stabilising selection in contrast to the meningococcus^94^.

**Figure 2.**
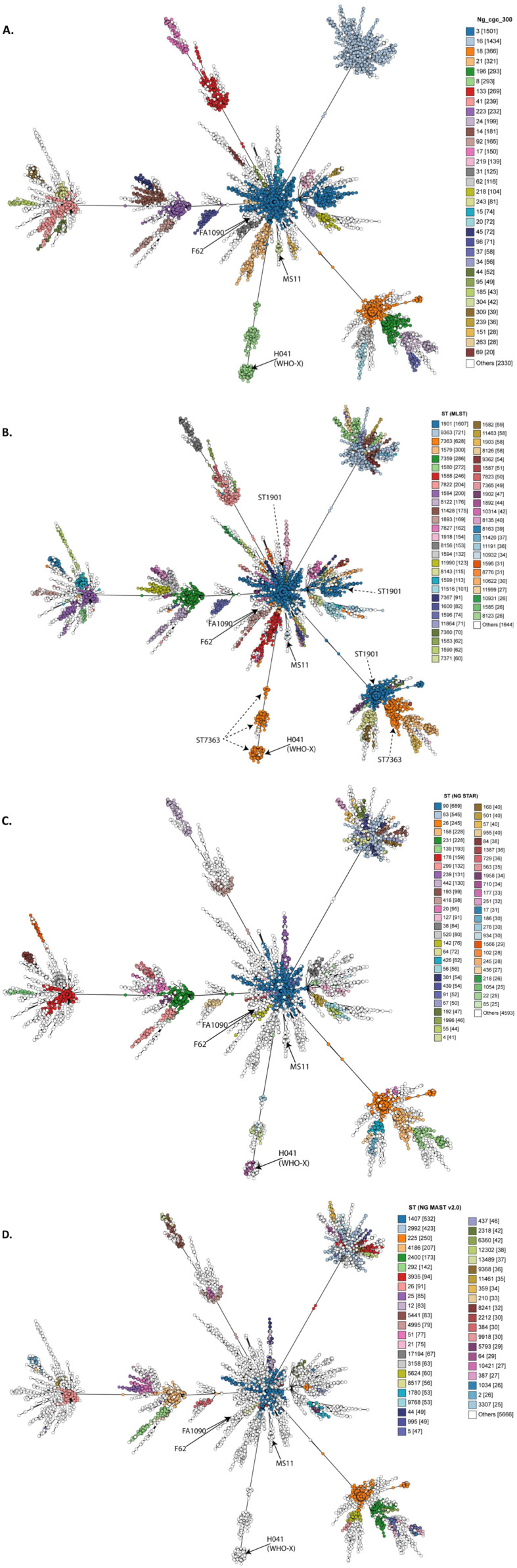
Minimum spanning trees comparing gonococcal whole genome sequence (WGS) using the core genome. In total, 9206 gonococcal WGSs were compared using the core genome (*Neisseria gonorrhoeae* cgMLST) and visualised with minimum spanning trees generated using Grapetree^84,102^. Isolates are clustered on the basis of similarities in allelic profiles in the core genome. (**A**) Nodes are coloured by core genome group using the 300 locus differences or fewer threshold. (**B**) Multi-locus sequence typing sequence type (MLST ST). (**C**) *N. gonorrhoeae* sequence typing for antimicrobial resistance type (NG STAR)^88^. (**D**) *Neisseria gonorrhoeae* multi-antigen sequence typing scheme (NG MAST ST v2.0)^82^. Gonococci possessing the same MLST ST while having distinct core genome content are evident, particularly for MLST ST-1901 (indicated by dark blue closed circles) and ST-7363 (dark orange and dashed arrows). This visualisation also makes associations between core genome and NG STAR STs apparent. Numbers in brackets refer to the number of isolates belonging to that group. Legends depict only groups containing 10 to 20 (or more) isolates. Commonly used laboratory strains FA1090, F62 and MS11 are indicated as well as the resistant strain H041 first identified in Japan.

The high rate of HGT observed in the gonococcus led to the assumption that limited structure would be evident in its populations^95^. Genomic population studies have shown otherwise^96,97^. Hierarchical Bayesian analyses (Bayesian Analysis of Population Structure, or BAPS) uses nucleotide polymorphism–based alignments to generate clusters of related isolates after accounting for HGT identified by tools such as Gubbins^98,99^. With this method, gonococcal genome data from several studies have been found to consist of between six and 12 BAPS clusters, some of which associated with AMR phenotypes^96,97,100^. This is consistent with a semi-clonal population structure (i.e., one in which there is residual clonal signal in the population)^96,97,100,101^. The presence of distinct gonococcal lineages was also evident in studies of the gonococcal core genome. For example, more than 1,600 genes were identified as ‘core’ in WGS data from over 4,000 gonococci: these genes have been defined in the PubMLST database (https://pubmlst.org/neisseria) and form part of the *N. gonorrhoeae* cgMLST version 1.0 scheme^84^. Core genome STs are assigned for each isolate and, through single-linkage** clustering and the use of increasing allelic difference thresholds, isolates can be grouped into related core genome groups (Figure 2). Use of this approach identified discrete clusters of gonococci, some of which persisted over time and associated with AMR genotypes^84^. Thus, analyses composed of higher numbers of genes, and therefore higher genome content, improve resolution and are required to investigate the gonococcal population and detect structure in this highly recombining species.

In the meningococcus, non-overlapping antigenic repertoires are found among different genotypes, resulting in the circulation of distinct meningococcal lineages, the prevalence of which fluctuates over time, probably as a consequence of changing natural immunity or vaccination or both^103–105^. However, it is apparent that other modes of selection are exerted on the gonococcal population, and AMR is a major influencing factor^96^. This was shown in the high prevalence of plasmid-mediated AMR in gonococci originating from low- and middle-income countries where, more often than not, syndromic management of gonorrhoea, including treatment with doxycycline, exists^106^. As a result, positive selection of gonococci harbouring conjugative plasmids expressing TetM occurs, resulting in the high prevalence of plasmid-mediated AMR^44^. That study also identified an association between plasmid types and the core genome, providing further evidence for the presence of structure within the gonococcal population.

The availability of WGS has had a major impact in enhancing our understanding of the population biology of the gonococcus and provides hope that it may be possible in the near future to curb transmission through the identification of gonococcal lineages circulating globally. In addition, the identification of loci constituting the core genome, which are common to all gonococci, provides an opportunity to assess antigenic diversity across the gonococcal population, equipping us with novel insights in vaccine development.

## Vaccine development

The capacity of the gonococcus to become resistant to chemotherapy provides the alarming prospect that this infection will become untreatable in the near future. This has led to a renewed interest in preventing infection through immunisation and vaccine development. However, despite several decades of research in this field, no vaccine has yet been successfully developed for use in humans^107^.

Reasons for the difficulties encountered in gonococcal vaccine development stem from a combination of characteristics exhibited by the gonococcus that facilitate immune evasion^9^. A major challenge is the fact that the gonococcus causes infections solely in humans and it has been difficult to develop animal models of infection in which immune responses and vaccines can be evaluated^107^. However, improvements made in mouse models of infection, including BALB/C, C57BL/6, and genetically engineered mice, have provided insight into the complexities of pathogenesis and the host response elicited^107^. For example, the lack of protective immunity elicited following natural infection may be a consequence of the ability for gonococci to subvert host immune responses^108^. Indeed, studies of experimentally infected female mice indicate that gonococcal infection of the genital tract results in suppression of adaptive Th1- and Th2-governed responses and induction of Th17-driven innate responses, which the gonococcus is able to resist^107,109,110^.

Immune evasion is also facilitated by the ability of gonococci to exhibit extensive antigenic diversity in surface-exposed antigens, a consequence of mutation, HGT, and the modulation of expression through phase variation^107^. As a result, appreciable variability will be present both among gonococci and within the same gonococcus over time. For example, intra-chromosomal recombination of promoterless copies of *pilS* that consist of the variable regions of the complete (expressed) *pilE* gene, which encodes the major pilus subunit (pilin), provides a silent catalogue of sequences that can be reassorted into an expression locus to generate new *pilE* variants^111^. *N. gonorrhoeae* is also highly competent for genetic transformation, a process facilitated by a type IV secretion system which is present in over 90% of gonococci and which, in a contact-independent manner, secretes single-stranded DNA into the environment, providing a source of DNA for HGT^112^.

In spite of these challenges, several *N. gonorrhoeae* antigens, some of which are promising vaccine candidates based on their antigenic conservation, distribution in gonococcal populations, and immunogenicity, have been characterised (Table 2). Several of these antigens have been identified using a combination of bioinformatic and proteomic analyses, which provide novel means of identifying vaccine antigens that would not have been detected using more conventional methods^113^. Indeed, through the use of proteomics-driven reverse vaccinology, the quantitative proteomic analysis of cell envelopes and naturally occurring vesicles has led to the discovery of several additional vaccine candidates, including BamA, LptD, TamA, NGO2054 and NGO2139^114^, sparking renewed interest in vaccine development (Table 2)^113^. However, it is worth noting that a combination of techniques, methods and tools will be required to evaluate vaccine candidates effectively: (i) proteomics to identify the relative abundance, post-translational modifications and protein–protein interactions of those vaccine candidates; (ii) genomics to assess the genetic diversity, prevalence and distribution in gonococcal populations; and (iii) animal models to examine *in vivo* host responses and the potential for an immune response to be elicited.

**Table 2.** *Neisseria gonorrhoeae* vaccine candidates.

Gene	Product	*N. gonorrhoeae* FA1090 nomenclature (NGO)	*N. meningitidis* MC58 nomenclature (NMB)	Nomenclature in PubMLST (NEIS)	Notes	Included in the gonococcal core genome	References
*ACP*	Neisserial adhesion complex	NGO1981	NMB2095	NEIS2075	Found in the outer membrane of all *Neisseria* species; conserved surface-exposed lysozyme inhibitor. Two alleles identified in gonococci.	Yes	123
*aniA*	Nitrate reductase	NGO1276	NA (generally not expressed in meningococci)	NEIS2779	Essential for growth and survival of the gonococcus in oxygen-limiting conditions and shown to elicit an immune response.	Yes	124,125
*bamA* (previously known as *omp*85)	Membrane biogenesis	NGO1801	NMB0182	NEIS0173	Part of the beta-barrel assembly machinery (BAM). BamA consists of a beta-barrel outer membrane protein with five repeated polypeptide transport-associated (POTRA) domains that extend into the periplasm.	Yes	126
*lbpA/lbpB*	Lactoferrin binding proteins	Not defined in FA1090	NMB1540/ NMB1541	NEIS1468/ NEIS1469	*lbpA* expression is phase-variable in the gonococcus.	Yes	127
*lptD* (also called *ostA*)	LPS assembly protein; outer membrane beta- barrel protein	NGO1715	NMB0280	NEIS0275	Together with LptE, LptD is involved in the assembly of lipopolysaccharide (LPS) at the surface of the outer membrane. Found to be essential for gonococcal viability.	Yes	113
*metQ*	Methionine receptor	NGO2139	NMB1946	NEIS1917	Surface-expressed antigen eliciting bactericidal and functionally blocking antibodies; putative lipoprotein.	Yes	113,128,129
*mtrE*	Outer membrane channel of the MtrCDE efflux pump	NGO1363	NMB1714	NEIS1632	Highly conserved with in particular loop 2 eliciting protein-specific antibodies.	Yes	9,130
*nspA*	Outer membrane beta-barrel protein	NGO0233	NMB0663	NEIS0612	Neisseria surface protein A which binds human factor H. Antibodies elicited by *N.* *meningitidis* NspA have been found to be bactericidal.	Yes	131
*opcA*	Class 5 Outer membrane protein	NGO0868	NMB1053	NEIS2198	Essential for adherence with antibodies elicited to OpcA found to block interactions with the host.	Yes	120,132
*pilQ*	Type IV pilus biogenesis and competence protein; pilus secretin.	NGO0094	NMB1812	NEIS0408	Essential for pilus function. Promising as antibodies against meningococcal PilQ are bactericidal.	Yes	132
*porB*	Outer membrane protein	NGO1812	NMB2039	NEIS2020	Constitutively expressed outer membrane protein. Two variants present in gonococci (PIA and PIB) with extensive diversity found, particularly in the surface-exposed loops of which there are eight.	Yes	133
*pld*	Phospholipase D	NGO0902	NMB1434	NEIS1368	PLD phosphodiesterase; putatively implicated in membrane ruffling leading to cellular invasion and facilitating survival *in* *vivo.*	Yes	134,135
*sliC*	Surface-exposed lysozyme inhibitor of c-type lysozyme	NGO1063	NMB1490	NEIS1425	Provides protection against the bactericidal effects of host lysozymes.	Yes	136
*tamA* (Omp85 family protein)	Translocation assembly machinery (TAM) consisting of *tamA* and *tamB* genes	NGO1956 (*tamA*) NGO1955 (*tamB*)	NMB2134 (*tamA*) NMB2135 (*tamB*)	NEIS2112 (*tamA*) NEIS2113 (*tamB*)	Part of the translocation and assembly module (TAM) autotransporter assembly complex, which functions in translocation of autotransporters across the outer membrane.	Yes	126
*tbpA/tbpB*	Transferrin binding proteins	NGO1495 (*tbpA*) NGO1496 (*tbpB*)	NMB0461/ NMB0460	NEIS1690/ NEIS1691	Consisting of two proteins expressed in iron- restricted environments. Extensive diversity present, particularly in TbpB.	Yes	137
*znuD*	Zinc receptor/ uptake component D	NGO1205	NMB0964	NEIS0944	Putative TonB-dependent outer membrane protein expressed under zinc limitation; ZnuD-specific antibodies detected in sera from patients convalescent from meningococcal disease.	Yes	120,138,139
*tuf*	Elongation factor Ef-Tu	NGO1842 and NGO1858	NA	NEIS0116 and NEIS0128	Identified in proteomics profiling of cell envelopes and outer membrane vesicles (OMVs) from *N. gonorrhoeae* strains. EF-Tu is a cytosolic GTP binding protein essential factor in protein synthesis. EF-Tu is encoded by at least two genes and has been identified previously in gonococcal periplasm as well as on the surface of bacterial cell envelopes and OMVs. Two copies of the *tuf* gene are present in *Neisseria* genomes.	Yes	108
*potF3*	Putrescine binding periplasmic protein	NGO1494	NA	NEIS1689	Part of the polyamine transport system consisting of the PA transporter genes *potFGHI.*	Yes	108,140
2C7 epitope of lipooligosaccharide core expressed through the action of the phase-variable *lgtG* gene	Lipooligosaccharide glycosyl transferase G	NGO1715	NA	NEIS2011	Highly immunogenic in human gonococcal infection. Although the 2C7 epitope is phase-variable, under control of the *lgtG* glycosyltransferase, the 2C7 epitope has been found to be expressed *in vivo* in more than 95% of gonococci. Expression of this epitope promotes survival of the gonococcus in mouse experimental models (41).	No	141–143
*yraP* (GNA2091)	Part of an operon	NGO1985 (NGO1987, NGO1986 and NGO1984)	NMB2091 (NMB2089, NMB2090 and NMB2092)	NEIS2071 (NEIS2069, NEIS2070 and NEIS2072)	This gene is associated with conferring outer membrane stability and virulence in the meningococcus and is a component of the 4CMenB vaccine.	Yes	144

It is likely that vaccines containing a ‘cocktail’ of antigens will be required in order to generate broad protection against the *N. gonorrhoeae* population. In this respect, outer membrane vesicle (OMV) vaccines may be suitable since they contain many of the gonococcal surface antigens in their natural conformation^115,116^. Support for the use of OMV as vaccines comes with the epidemiological evidence provided by a retrospective case control study undertaken in New Zealand, which suggested that individuals vaccinated with the OMV vaccine MeNZB were less likely to contract gonorrhoea^117^. The MeNZB vaccine had been specifically designed in response to a serogroup B *N. meningitidis* epidemic in New Zealand, and although vaccine efficacy towards gonorrhoea was estimated to be 31%, ensuing studies have shown that cross-reactive anti-gonococcal antibodies are induced by MeNZB OMV proteins, providing hope that a gonococcal OMV vaccine is achievable^118^.

A vaccine targeting gonococci is now a possibility with the availability of WGS allowing an *in silico* appraisal of vaccine candidates to be undertaken prior to more costly and time-consuming vaccine development approaches. In particular, the gene-by-gene characterisation of the gonococcal core genome available on PubMLST allows the diversity of putative vaccine candidates to be catalogued across global gonococcal populations spanning decades^84,119,120^.

## Conclusions

Gonorrhoea has been long considered to be an ancient human disease on the basis of clinical descriptions, but recent research has suggested that the emergence of gonorrhoea may date back to as recently as the sixteenth century, calculated through the comparison of genome sequence data and through inference of the time to most recent common ancestor^96^. However, it is conceivable that a much longer association, potentially spanning millennia and consistent with descriptions in ancients texts, has existed^121^.

In any case, the coexistence of humans and gonococci has had, and will continue to have, a profound effect on both populations. In humans, effects include reduced birth rates resulting from infertility caused by ascending gonococcal infections, in addition to increased mortality rates through coinfection with HIV^122^. The coexistence of gonococci with humans has led to the development of multiple mechanisms of genetic and antigenic variation, allowing adaptation, persistence and evasion of both environmental and host challenges. In addition, it is most likely that human migration has widened the gonococcal gene pool with increased diversity as a result^145^. In more recent times, antimicrobial use has further shaped the gonococcal population, leading to the expansion of AMR lineages^84,96^.

Genomics plays an increasingly important role in combatting gonococcal disease, allowing the definition and characterisation of complex characters, including the genetic elements associated with AMR, persistence and antigenic diversity. However, at the time of writing, only a very small proportion of gonococci causing infection were analysed by WGS. For example, 56,259 infections were diagnosed in England in 2018 and it would be both expensive and impractical to sequence or culture all of these in one year using current sequencing platforms. Therefore, a large proportion of the *N. gonorrhoeae* isolates circulating globally will remain uncharacterised at the WGS level for the near future. Our current understanding will be based on a subset of the gonococcal population and at the time of writing this was strongly biased towards isolates with an AMR phenotype. To further our ability to cure or prevent gonorrhoea, larger and more representative datasets need to be assembled and sequenced. Sequencing technology has made major advances in the last decade and what seems impossible now may be achievable in the future. Until then, it is likely that the gonococcus will continue to elude us.

## Abbreviations

AMR, antimicrobial resistance; BAPS, Bayesian Analysis of Population Structure; cc, clonal complex; ESC, extended-spectrum cephalosporin; EU/EEA, European Union/European Economic Area; HGT, horizontal gene transfer; MDR-GC, multidrug-resistant gonococci; MIC, minimum inhibition concentration; MLST, multi-locus sequence typing; NG MAST, *Neisseria gonorrhoeae* multi-antigen sequence typing; NG STAR, *Neisseria gonorrhoeae* sequence typing for antimicrobial resistance; OMV, outer membrane vesicle; ST, sequence type; XDR-GC, extensively drug-resistant gonococci; WGS, whole genome sequencing
